# Exploring dimensions of social capital in relation to healthy eating behaviours in the US rural south

**DOI:** 10.1017/S1368980023000022

**Published:** 2023-05

**Authors:** Cerra C Antonacci, Regine Haardörfer, April K Hermstad, Tilicia L Mayo-Gamble, Kimberly R Jacob Arriola, Michelle C Kegler

**Affiliations:** 1 Department of Behavioral, Social and Health Education Sciences, Emory Prevention Research Center, Rollins School of Public Health, Emory University, Atlanta, GA 30322, USA; 2 Department of Health Policy and Community Health, Georgia Southern University, Statesboro, GA, USA

**Keywords:** Fruit and vegetable intakes, Sugar-sweetened beverages, Social capital, Rural adults

## Abstract

**Objective:**

This study examined relationships between dimensions of social capital (SC) (social trust, network diversity, social reciprocity and civic engagement) and fruit, vegetable, and sugar-sweetened beverage (SSB) consumption among rural adults. Potential moderators (neighbourhood rurality, food security, gender and race/ethnicity) were explored to develop a more nuanced understanding of the SC–healthy eating relationship.

**Design:**

Data were from a 2019 mailed population-based survey evaluating an eleven-county initiative to address health equity. Participants self-reported health behaviours, access to health-promoting resources and demographics. Logistic regression models were used to analyse relationships between predictors, outcomes and moderators.

**Setting:**

Five rural counties, Georgia, USA.

**Participants:**

1120 participants.

**Results:**

Among participants who lived in the country (as opposed to in town), greater network diversity was associated with consuming ≥ 3 servings of fruit (OR = 1·08; 95 % CI 1·01, 1·17, *P* = 0·029), yet among participants who lived in town, greater civic engagement was associated with consuming ≥ three servings of fruit (OR = 1·36; 95 % CI 1·11, 1·65, *P* = 0·003). Both food-secure and food-insecure participants with greater social reciprocity had lower odds of consuming 0 SSB (OR = 0·92; 95 % CI 0·86, 0·98, *P* = 0·014, OR = 0·92; 95 % CI 0·86, 0·99, *P* = 0·037, respectively). Men with greater social trust were more likely to consume 0 SSB (OR = 1·09; 95 % CI 1·01, 1·18, *P* = 0·038), and Whites with greater network diversity were more likely to meet daily vegetable recommendations (OR = 1·10; 95 % CI 1·01, 1·19, *P* = 0·028).

**Conclusions:**

Findings provide a basis for future qualitative research on potential mechanisms through which SC and related social factors influence healthy eating in rural communities.

A healthy diet provides nutrients, facilitates weight management and reduces risk for diseases such as heart disease, stroke and some cancers^(1)^. The USDA’s 2020 Dietary Guidelines for Americans (DGA) recommends consuming at least 1·5 cups of fruit and 2 cups of vegetables per d and limiting added sugars^(1)^. While most American adults fail to meet the recommended daily servings of fruits and vegetables^(2)^, an even smaller proportion of Americans living in rural communities meet these recommendations^(3,4)^. Similarly, adults living in rural counties consume more sugar-sweetened beverages (SSB) compared with their urban and suburban counterparts^(5)^. These differences in healthy eating behaviours likely contribute to the fact that rural residence has been identified as an independent risk factor for heart disease, diabetes and obesity^(3,6)^.

Several studies also highlight social capital (SC) as an important contextual factor related to health. According to Putnam^(7)^, SC is a collective good, described by features of social organisation including the ‘connections among individuals – social networks and the social reciprocity and trustworthiness that arise from them’ (p. 18). SC is a multidimensional construct characterised by interrelated dimensions such as social trust, network diversity, social reciprocity, civic engagement and voting behaviour^(7)^. While some choose to measure SC using one or more dimensions^(8–11)^, others measure the whole construct by combining dimensions into one index^(12,13)^. SC is thought to create health-enabling resources that support health-promoting behaviours through the creation of social support networks and reinforcement of norms within a network^(7,14)^. For instance, in communities with high SC where residents practice healthy eating behaviours, other community residents may be encouraged to engage in those same health behaviours through the reinforcement of healthy eating norms and the social support available to them to maintain those behaviours^(14)^. Research has generally found that higher levels of SC are associated with healthier eating behaviours^(9,15)^. For instance, Poortinga^(9)^ measured several dimensions of SC (i.e. social support, civic engagement and social trust) and found that those SC dimensions were associated with consuming five or more portions of fruits and vegetables per d.

Like eating behaviours, SC also differs by place^(16,17)^. Rural areas are traditionally thought to have higher levels of SC because of the smaller, more tight-knit communities that exist in rural settings^(7)^. Research has supported this contention by finding that rural communities have greater localised trust, higher rates of civic engagement and more local reciprocity compared with urban areas^(16)^. While previous research has elucidated the relationship between SC, other health behaviours, and general health in the rural context^(18,19)^, and the relationship between SC and healthy eating in urban settings^(15)^, there is currently a lack of research exploring the relationship between SC and healthy eating behaviours in the rural setting. To our knowledge, only one study has investigated this relationship in a rural setting. Johnson and colleagues examined extra-familial social support as one dimension of SC and found that SC positively associated with fruit and vegetable consumption among rural adults in Texas^(8)^.

The purpose of this research is to gain a deeper understanding of the relationship between SC dimensions and healthy eating behaviours among residents of rural counties. While previous research has examined many dimensions of SC^(8–12,15,16,20)^, most research focuses on an average of two dimensions^(21)^. We analyse four dimensions of SC to gain a more comprehensive picture of SC and explore how those dimensions relate to three eating behaviours. We hypothesise that higher social trust, network diversity, social reciprocity and civic engagement will be associated with higher fruit and vegetable consumptions and lower SSB consumption (see conceptual model in Fig. 1). Additionally, previous research has independently explored variations in SC and healthy eating behaviours by different contextual (e.g. urbanicity/rurality and food security) and demographic factors (e.g. gender and race/ethnicity)^(2,5,7,22–24)^, but there is a lack of research on the moderating roles of such variables on the SC–healthy eating relationship. We explore whether and how perceived neighbourhood rurality, food security, gender and race/ethnicity impact the SC–healthy eating relationship.

**Fig. 1:**
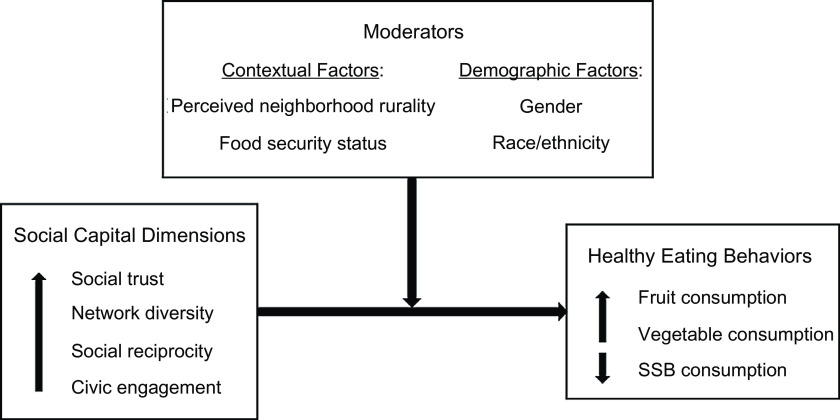
Conceptual model of the relationship between dimensions of social capital and healthy eating behaviours

Studies suggest that SC increases when moving from urban to rural areas^(7,11,12)^. These studies, however, typically focus on urban, semi-urban and rural differentiations, on objective classifications of rurality, and county-level classifications (e.g. rural–urban continuum codes), rather than perceived rurality at the neighbourhood level (e.g. in town *v*. in the country), which may relate to how connected people feel to their communities. Similarly, food insecurity (i.e. the lack of access to sufficient food to live a healthy life)^(4)^ is more pervasive in rural areas^(25)^, likely due to food access issues (e.g. farther travel distances to grocery stores and higher food prices)^(26)^ and has been found to associate with SC and eating behaviour^(4,5,13,27,28)^. Food insecurity is thought to relate to SC through knowledge and product sharing, impacting both access to and availability of food^(13)^. Food insecurity has inversely associated with SC in studies of different populations, including rural adults^(4,13)^, and has associated with lower fruit and vegetable consumption^(28)^ and greater SSB consumption among adults in rural and urban areas^(5)^. We hypothesise that the relationships between SC dimensions and healthy eating behaviours will be stronger for those who self-reported living in the country compared with those who self-reported living in town and for food-secure (FS) compared with food-insecure (FI) adults.

SC and healthy eating are also independently associated with gender and race/ethnicity. Compared with men, women are more likely to meet the daily recommended servings of fruits and vegetables^(2)^ and consume fewer SSB^(5)^, and African American/Blacks (AA/Black) tend to consume fewer vegetables but more fruits and SSB compared with Whites^(2,22,29)^. SC also varies by gender, though without a clear pattern^(10,23)^, and by race/ethnicity^(24)^, with one publication asserting the negative effects of SC in perpetuating inequalities in workplace hiring that favour White men over women and minorities^(30)^. We hypothesise that gender will impact the relationship between SC and healthy eating differently depending on the SC dimension, and we expect the strength of the SC–healthy eating relationships will be stronger for Whites compared with AA/Blacks.

## Methods

### Study design and sample

The data for this study were from an evaluation of *The Two Georgias Initiative* (TGI), a multi-year initiative developed by Healthcare Georgia Foundation to support eleven community coalitions in rural Georgia counties (i.e. those with populations < 35 000) with the goals of achieving health equity, improving health and healthcare, building leadership capacity, and eliminating health disparities. As part of the evaluation, a population-based survey was mailed to 11 406 randomly selected households within the eleven counties where the eleven coalitions were located between December 2018 and June 2019, followed by a reminder postcard and a second mailed survey as needed. 2788 completed surveys were returned (24·4 % response rate). The survey included modules on health behaviours, access to health-promoting resources (e.g. healthy foods, healthcare and SC) and demographic characteristics. As not all coalitions collected data on all topics, only those that administered both the SC and healthy eating modules were included in this study; five of the eleven counties completed modules relevant to these analyses resulting in a subsample of 1120 adults. Per the Emory University Institutional Review Board (IRB), this study did not require IRB approval because it was non-research programme evaluation.

### Measures

#### Healthy eating behaviours

Healthy eating behaviours were measured by daily fruit, vegetable and SSB consumption. Daily fruit and vegetable intakes were measured using a previously validated two-item measure^(4,31)^ that asked participants to report the number of servings of fruits and vegetables they typically eat each day. The fruit and vegetable variables were dichotomised into ‘met’ or ‘did not meet’ the 2020 DGA recommendations, which suggests consuming at least 2 cups of vegetables and 1·5 cups of fruit per d^(1)^. As the DGA recommendations are based on cups and our survey asked about servings, we converted DGA recommendations from cups into servings (i.e. ½ cup = 1 serving)^(32)^; following this conversion method, the number of recommended servings of fruits and vegetables were three and four servings, respectively. A measure for daily intake of 12-ounce units of SSB, including sweet tea, regular sodas, bottled fruit drinks, sports drinks and Kool-Aid, was developed for this survey and included six response options (0, 1, 2, 3, 4, 5, or more). This variable was dichotomised into consuming zero and one or more SSB per d to align with CDC’s reporting of data from the Behavioral Risk Factor Surveillance System (BRFSS)^(33)^.

#### Social capital

SC was measured with twenty-nine items (*a* = 0·91) assessing four dimensions of SC (i.e. social trust, network diversity, social reciprocity and civic engagement). The social trust and network diversity questions were based on the Social Capital Benchmark Survey (SCBS)^(34)^; the social reciprocity questions were based on an instrument designed and validated by Sampson et al.^(35,36)^; most of civic engagement questions were based on the SCBS^(34)^, while the remaining items were taken from the Community Population Survey-Civic Engagement Supplement^(37)^. Eight social trust items^(34)^ were included and asked participants to report their level of trust in different groups (people in general, friends and family, people that live near you, people from a different racial/ethnic group than your own, people with different political views than your own, people from a different religion than your own, people with more or less education than you, and people with a lot more or a lot less money than you) with four response options ranging from trust them a lot (3 points), trust them some, trust them only a little and trust them not at all (0 points). Responses were summed to create a social trust index ranging from 0 to 24. To measure network diversity, participants were asked to indicate how often they talk to or spend time with the same eight groups of people in the social trust index with four response options ranging from very often (3 points), often, not very often and not at all (0 points)^(34)^. Responses were summed to create a network diversity index ranging from 0 to 24. Three questions were included to measure social reciprocity^(35,36)^: ‘People around here are willing to help their neighbors’, ‘If I had to borrow $30 in an emergency, I could borrow it from a neighbor’ and ‘If I were sick, I could count on my neighbors to shop for groceries for me’. A five-point Likert scale was used to categorise the degree to which participants agreed with the statements, ranging from strongly disagree (1 point), disagree, neutral, agree and strongly agree (5 points). Responses were summed to create a social reciprocity index ranging from 3 to 15. Finally, ten yes/no (1/0 points) questions were related to civic engagement^(34,37)^ and asked participants about their past 12-month involvement in various activities (e.g. attend any club or organisational meeting). Responses were summed to create a civic engagement index ranging from 0 to 10.

#### Demographics

Demographic variables were self-reported and included age, level of completed education, annual household income, weight in pounds, and height in inches. BMI was constructed using height and weight data. Other demographic variables included the potential moderators of degree of rurality, food security status, gender and race/ethnicity. As a covariate, the original race/ethnicity variable was used as is; however, for bivariate and multivariable analyses, the variable was dichotomised into White and AA/Black. Perceived neighbourhood rurality was measured with one item offering three answer choices (in town, in the country with neighbours close by and in the country with very few neighbours)^(38)^ and was dichotomised into living in town or living in the country. A previously validated two-item measure was used to assess food security status^(39)^. Participants were asked to indicate never, sometimes or often as to whether two scenarios affected them in the previous 12 months: ‘I worried whether my food would run out before I got money to buy more’ and ‘The food I bought just didn’t last and I didn’t have money to get more’. Those who responded sometimes or often to at least one of the statements were classified as FI.

### Analysis

Descriptive statistics were run on sample characteristics. Continuous variables were described with means and standard deviations, while categorical variables were described using frequencies and percentages. Bivariate analyses assessed associations between all four SC variables and the four moderators using ANOVA, the three healthy eating behaviours and the SC variables using ANOVA, and the healthy eating behaviours and moderators using chi-squared tests.

Separate multivariable logistic models were developed to examine associations between SC dimensions and the probability of meeting DGA recommendations for each of the healthy eating behaviours (≥ 3 servings of fruit, ≥ 4 servings of vegetables and 0 12-ounce SSB), as well as possible moderation. Potential moderators were perceived neighbourhood rurality (in town and in the country), food security status (FS and FI), gender (male and female), and race/ethnicity (White and AA/Black). Initial models included the SC dimensions and interactions between the SC dimensions and moderators. Models were then pared down to include only significant interactions. Race, gender, age, income and education were added as covariates, and the county variable was added as a covariate to all models using indicator variables to adjust for clustering of the data by county. All analyses were conducted in SAS 9.4. Statistical significance was assessed at alpha = 0·05.

## Results

### Study participants

Sample characteristics are presented in Table 1. Among the 1120 participants, most were older, women, White, overweight or obese, and FI, and had low educational attainments and yearly incomes. Only 11·2 % of participants met the DGA recommendation for fruit, even fewer (9·3 %) met the DGA recommendation for vegetables, and approximately one-third of participants did not consume any SSB per d.

**Table 1:** Sample characteristics

Variable	Mean	sd	*n*	%
Counties				
County 1			196	17·5
County 2			239	21·3
County 3			176	15·7
County 4			219	19·6
County 5			290	25·9
Demographics				
Age (years)	59·7	15·38		
Gender* (women)			735	66·5
Race/ethnicity*				
White			804	71·9
African American/Black, not of Hispanic origin			251	22·4
More than one race			15	1·3
Other			10	0·9
Hispanic			7	0·6
Household size	2·3	1·46		
Perceived neighbourhood rurality* (in town)			347	31·3
BMI (kg/m^2^)				
Underweight (< 18·5)			11	1·0
Normal (18·5–24·9)			273	24·7
Overweight (25·0–29·9)			359	32·5
Obese (≥ 30)			460	41·7
Socio-economic status				
Education				
Some high school or less			116	10·6
High school or GED			304	27·7
Some college or technical school			348	31·7
College and above			331	30·1
Annual household income				
< $20 000			263	28·9
$20 000–$50 000			331	36·4
> $50 000			315	34·7
Food security* (food-insecure)			430	46·1
Social environment				
Social capital†				
Social trust	14·5	4·90		
Network diversity	13·7	4·81		
Social reciprocity	10·2	3·39		
Civic engagement	3·3	2·78		
Eating behaviour				
Fruit intake (≥ 3 servings/d)			125	11·2
Vegetable intake (≥ 4 servings/d)			104	9·3
SSB intake (0 12 oz SSB/d)			400	36·2

SSB, sugar-sweetened beverages.

* Potential moderators.

† Possible ranges for social capital indexes: social trust (0–24), network diversity (0–24), social reciprocity (3–15) and civic engagement (0–10).

### Bivariate associations

Regarding the bivariate associations between the SC dimensions and the moderators (Table 2), compared with people who lived in town, those who lived in the country had higher levels of social trust (M_country_ = 14·7, sd_country_ = 4·62, M_town_ = 13·6, sd_town_ = 5·47, *P* = 0·023) and social reciprocity (M_country_ = 10·4, sd_country_ = 3·28, M_town_ = 9·7, sd_town_ = 3·56, *P* = 0·001). FS participants, compared with those who were FI, had higher levels of all SC dimensions. Compared with women, men had higher levels of social trust (M_men_ = 15·1, sd_men_ = 4·45, M_women_ = 14·2, sd_women_ = 5·08, *P* = 0·009), social reciprocity (*M*_*men*_ = 10·7, sd_*men*_ = 3·14, *M*_*women*_ = 10·0, sd_*women*_ = 3·47, *P* = 0·001) and civic engagement (*M*_*men*_ = 3·6, sd_*men*_ = 2·85, *M*_*women*_ = 3·1, sd_*women*_ = 2·71, *P* = 0·004). Similarly, Whites had higher levels of all SC dimensions compared with AA/Blacks.

**Table 2:** Bivariate associations between SC dimensions and potential moderators†

Variable	Social trust			Network diversity			Social reciprocity			Civic engagement		
	Mean	sd	*P*	Mean	sd	*P*	Mean	sd	*P*	Mean	sd	*P*
Perceived neighbourhood rurality												
In town	13·95	5·47	0·023*	13·60	5·11	0·878	9·68	3·56	0·001*	3·24	2·73	0·879
In the country	14·71	4·62		13·65	4·68		10·42	3·28		3·27	2·79	
Food security status												
Food-secure	16·01	4·47	< 0·0001*	14·66	4·47	< 0·0001*	11·39	2·77	< 0·0001*	3·78	2·73	< 0·0001*
Food-insecure	12·74	4·85		12·48	4·92		8·64	3·38		2·40	2·53	
Gender												
Male	15·05	4·45	0·009*	13·98	4·45	0·147	10·70	3·14	0·001*	3·61	2·85	0·004*
Female	14·20	5·08		13·52	4·97		9·95	3·47		3·08	2·71	
Race/ethnicity												
White	15·41	4·51	< 0·0001*	14·25	4·68	< 0·0001*	10·74	3·19	< 0·0001*	3·70	2·76	0·022*
AA/Black	11·73	4·70		11·81	4·65		8·64	3·64		2·88	2·75	

SC, social capital.

Associations run using PROC ANOVA.

* Significance at *P* < 0·05.

Table 3 displays the results of the bivariate associations between the healthy eating behaviours and SC dimensions and between the healthy eating behaviours and potential moderators. Participants who met or exceeded three servings of fruit per d had higher levels of social trust (M _≥ 3 servings_ = 15·3, sd_≥ 3 servings_ = 5·19, M _< 3 servings_ = 14·4, sd_< 3 servings_ = 4·86, *P* = 0·045), network diversity (M _≥ 3 servings_ = 14·8, sd_≥ 3 servings_ = 4·76, M _< 3 servings_ = 13·5, sd_< 3 servings_ = 4·8, *P* = 0·008) and social reciprocity (M _≥ 3 servings_ = 10·8, sd_≥ 3 servings_ = 3·17, M _< 3 servings_ = 10·1, sd_< 3 servings_ = 3·41, *P* = 0·040) compared with those who consumed fewer than three daily servings of fruit (Table 3). Compared with those who did not meet the recommendation of eating four daily servings of vegetables, participants who met the recommendation had higher levels of network diversity (M _≥ 4 servings_ = 14·8, sd_≥ 4 serving_ = 4·97, M _< 4 servings_ = 13·5, sd_< 4 servings_ = 4·77, *P* = 0·010) and civic engagement (M _≥ 4 servings_ = 4·0, sd_≥ 4 servings_ = 2·71, M _< 4 servings_ = 3·2, sd_< 4 servings_ = 2·77, *P* = 0·005). Participants who consumed zero daily servings of 12-ounce SSB experienced higher levels of all SC dimensions.

**Table 3:** Bivariate associations between healthy eating behaviours and SC dimensions and between healthy eating behaviours and moderators

Variable	Fruit consumption					Vegetable consumption					SSB consumption				
	≥ 3 servings per d			< 3 servings per d		≥ 4 servings per d			< 4 servings per d		0 servings per d			≥ 1 serving per d	
SC dimensions†	Mean	sd	*P*	Mean	sd	Mean	sd	*P*	Mean	sd	Mean	sd	*P*	Mean	sd
Social trust	15·32	5·19	14·35	4·86	0·045*	15·14	4·83	14·39	4·91	0·159	15·19	4·89	14·03	4·86	0·001*
Network diversity	14·77	4·76	13·51	4·80	0·008*	14·84	4·97	13·53	4·77	0·010*	14·23	4·75	13·33	4·78	0·004*
Social Reciprocity	10·80	3·17	10·12	3·41	0·040*	10·69	3·25	10·15	3·40	0·127	10·79	3·14	9·87	3·48	< 0·0001*
Civic engagement	3·68	2·84	3·21	2·76	0·081	4·03	2·71	3·18	2·77	0·005*	3·59	2·70	3·05	2·78	0·003*
Moderators‡	*n*	%	*n*	%	*P*	*n*	%	*n*	%	*P*	*n*	%	*n*	%	*P*
Perceived neighbourhood rurality															
In town	33	9·5	314	90·5	0·258	24	6·9	323	93·1	0·076	130	38·2	210	61·8	0·354
In the country	90	11·8	672	88·2		78	10·2	684	89·8		266	35·3	487	64·7	
Food security status															
Food-secure	74	14·7	428	85·3	0·001*	61	12·2	441	87·9	0·004*	225	45·2	273	54·8	< 0·0001*
Food-insecure	32	7·4	398	92·6		28	6·5	402	93·5		112	26·6	309	73·4	
Gender															
Male	36	9·9	327	90·1	0·343	30	8·3	333	91·7	0·411	117	32·4	244	67·6	0·043*
Female	87	11·8	648	88·2		72	9·8	663	90·2		279	38·7	442	61·3	
Race/ethnicity															
White	98	12·2	706	87·8	0·027*	80	9·9	724	90·1	0·129	336	42·2	460	57·8	< 0·0001*
AA/Black	18	7·2	233	22·1		17	6·8	234	93·2		43	17·0	200	82·3	

SC, social capital. SSB, sugar-sweetened beverages.

* Significance at *P* < 0·05.

† Associations between SC indexes and eating behaviours were run using PROC ANOVA.

‡ Associations between moderators and eating behaviours were analysed with chi-squared in PROC FREQ.

Regarding the associations among the potential moderators and healthy eating behaviours (Table 3), there were no significant differences between perceived neighbourhood rurality and any of the healthy eating behaviours. In terms of food security status, compared with FI participants, FS participants were more likely to meet daily fruit recommendations [14·7 % of FS, 7·4 % of FI; *X* ^2^(1, 932) = 12·24, *P* = 0·001], meet daily vegetable recommendations [12·2 % of FS, 6·5 % of FI; *X* ^2^(1, 932) = 8·53, *P* = 0·004] and consume zero SSB per d [45·2 % of FS, 26·6 % of FI; *X* ^2^(1, 919) = 33·90, *P* < 0·0001]. Men were more likely to consume at least one SSB per d compared with women [67·6 % of men, 61·3 % of women; *X* ^2^(1, 1082) = 4·10, *P* = 0·043]. Lastly, in regard to race/ethnicity, a significantly greater percentage of White compared with AA/Black participants met daily fruit recommendations [12·2 % of Whites, 7·2 % of AA/Blacks; *X* ^2^(1, 1055) = 4·92, *P* = 0·027] and consumed zero SSB [42·2 % of Whites, 17·0 % of AA/Blacks; *X* ^2^(1, 1039) = 48·28, *P* < 0·0001].

### Multivariable associations

Three adjusted logistic regression models were run for the healthy eating behaviours to assess the relative contributions of the four SC dimensions and to analyse significant interactions among the moderators and SC dimensions (Table 4). There were no significant main effects in any of the models, though several significant interactions emerged.

**Table 4:** Adjusted OR for the main effect associations between SC dimensions and healthy eating behaviours†

Variable	Fruit consumption			Vegetable consumption			SSB consumption		
	Adjusted OR	95 % CI	*P*	Adjusted OR	95 % CI	*P*	Adjusted OR	95 % CI	*P*
Social trust	1·06	0·98, 1·14	0·168	0·99	0·91, 1·07	0·729	–		–
Network diversity	–		–	–		–	1·00	0·96, 1·05	0·930
Social reciprocity	0·98	0·90, 1·08	0·708	1·01	0·91, 1·13	0·796	–		–
Civic engagement	–		–	–		–	0·97	0·90, 1·05	0·493

SC, social capital. SSB, sugar-sweetened beverages; DGA, Dietary Guidelines for Americans.

† Regressions were run using PROC LOGISTIC and modelled the probability of meeting DGA recommendations for fruits and vegetables, and for consuming 0 12-ounce SSB. Main effects not reported for predictors involved with significant interactions. Adjusted for age, sex, gender, race, income, level of education and county clustering.

Perceived neighbourhood rurality moderated two relationships related to fruit consumption and SC (Table 5). The relationship between fruit consumption and network diversity was moderated by perceived neighbourhood rurality but only among those who lived in the country [OR = 1·08; 95 % CI 1·01, 1·17, *P* = 0·029]. The relationship between fruit consumption and civic engagement was also moderated by perceived neighbourhood rurality, such that those who lived in town had 36 % higher odds of meeting the DGA recommendation for fruit (95 % CI 1·11, 1·65, *P* = 0·003), but no relationship was found for those who lived in the country.

**Table 5:** Moderation results modelling the odds of consuming ≥ 3 servings of fruit per d, ≥ 4 servings of vegetables and 0 12-ounce SSB per d†

Consumed ≥ 3 servings of fruit						
	Adjusted OR	95 % CI	*P*	Adjusted OR	95 % CI	*P*
	In town			In the country		
Network diversity	0·95	0·86, 1·04	0·282	1·08	1·01, 1·17	0·029*
Civic engagement	1·36	1·11, 1·65	0·003*	0·95	0·83, 1·08	0·425

Consumed ≥ 4 servings of vegetables						
	Adjusted OR	95 % CI	*P*	Adjusted OR	95 % CI	*P*
	Food-secure			Food-insecure		
Civic engagement	1·07	0·93, 1·22	0·349	1·09	0·94, 1·24	0·250
	White			AA/Black		
Network diversity	1·10	1·01, 1·19	0·028*	0·90	0·77. 1·04	0·151

Consumed 0 12-ounce SSB						
	Adjusted OR	95 % CI	*P*	Adjusted OR	95 % CI	*P*
	Food-secure			Food-insecure		
Social reciprocity	0·92	0·86, 0·98	0·014*	0·92	0·86, 0·99	0·037*
	Male			Female		
Social trust	1·09	1·01, 1·18	0·038*	1·00	0·95, 1·06	0·994

SSB, sugar-sweetened beverages; DGA, Dietary Guidelines for Americans.

* Significance at *P* < 0·05.

† Regressions run in PROC LOGISTIC and modelled the probability of meeting DGA daily recommendations for fruits and vegetables, and for consuming 0 12-ounce SSB; Adjusted for age, sex, gender, race, income, level of education and county clustering.

Food security status moderated two relationships between healthy eating behaviours and SC (Table 5). The relationship between SSB consumption and social reciprocity was moderated by food security status. For every 1-unit increase in social reciprocity, both FS and FI participants had lower odds of consuming zero SSB (OR = 0·92; 95 % CI 0·86, 0·98, *P* = 0·014, and OR = 0·92; 95 % CI 0·86, 0·99, *P* = 0·037, respectively). The relationship between vegetable consumption and civic engagement was also moderated by food security status in the model testing of all potential interactions (*P* = 0·049); however, the relationship was no longer significant in the model with only significant interaction terms (*P* = 0·813), and no differences were found between those who were FS and those who were FI.

Gender and race/ethnicity each moderated one relationship (Table 5). Men had 9 % higher odds (95 % CI 1·01, 1·18, *P* = 0·038) of consuming zero SSB for every 1-unit increase in social trust; however, no relationship was found for women. Whites had 10 % higher odds of meeting the DGA recommendation for vegetables (95 % CI 1·01, 1·19, *P* = 0·028) for every 1-unit increase in network diversity, but the data did not support this relationship among AA/Blacks.

## Discussion

The main aim of this study was to explore whether four dimensions of SC were associated with three healthy eating behaviours and to investigate whether those relationships were moderated by perceived neighbourhood rurality, food security status, gender and race/ethnicity. The percentage of respondents meeting the DGA recommended servings of fruits and vegetables (11·2 % and 9·3 %, respectively) was similar to national estimates (12·2 % and 9·3 %, respectively) and estimates for the state of Georgia (12·0 % and 8·5 %, respectively)^(2)^. While the SC dimensions were associated with the healthy eating variables in the expected directions in bivariate analyses (see conceptual model in Fig. 1), there were no significant main effects between SC dimensions and healthy eating variables in regression models.

### Contextual factors related to social capital and healthy eating

Food insecurity was especially high in our sample with 46·1 % of respondents classified as FI, compared with 26·2 % of Georgia households estimated to be FI based on data from the 2017 BRFSS^(39)^. Consistent with the large body of research on food security and healthy eating behaviours^(13,27)^, our analyses highlight the strong positive relationship between being FS and having healthier eating behaviours, in the case of our data, consuming at least three servings of fruit, four servings of vegetables and zero 12-ounce servings of SSB per d. Further, and also consistent with previous research^(4,13,20)^, our results suggest a strong positive relationship between food security and all dimensions of SC. As SC is believed to reduce food insecurity through knowledge and product sharing, thus increasing one’s access to and availability of food^(13)^, it is possible that our data, though cross-sectional, are demonstrating that high levels of the different SC dimensions are protective against food insecurity through the exchange of information (e.g. food-related resources) and goods (e.g. food) among members of these rural communities. As far as food security status as a moderator, food security initially moderated two relationships, but only one remained significant in the model that included only significant interactions. Both FS and FI participants with higher amounts of social reciprocity were less likely to consume zero SSB. Of all the SC dimensions, social reciprocity (i.e. helping someone with the expectation that the same person will help you at some point in the future)^(16)^ is most aligned with the mechanism believed to drive the inverse relationship between SC and food insecurity (i.e. knowledge and product sharing). Perhaps our finding, that regardless of food security status social reciprocity is associated with a greater likelihood of consuming at least one SSB per d, reveals that goods (regardless of their healthfulness) may be shared among community members. Our categorisation of the healthy eating variables may have made it easier to pick up on this finding for SSB than for fruit or vegetable consumptions; future research might instead examine these variables continuously.

Our findings related to perceived neighbourhood rurality are more challenging to interpret. In bivariate analyses, one’s perception of living in the country (compared with living in town) was associated with higher social trust and social reciprocity. Our findings are unique in that we explored one’s perceived neighbourhood rurality, and these simple analyses are consistent with previous findings related to objective measures of rurality, which have shown that SC increases when moving from the urban to the rural end of the population density spectrum^(7,16,17)^. Perceived neighbourhood rurality also moderated several relationships, though results diverged from our hypothesis. Participants who self-reported living in the country (but not those who self-reported living in town) were more likely to meet the DGA recommendation for fruit with higher levels of network diversity (i.e. time spent talking to or spending time with different groups of people). However, participants who self-reported living in town (but not those who self-reported living in the country) had higher odds of eating the recommended daily servings of fruit with higher levels of civic engagement. Previous research comparing SC’s effects in urban and rural areas has found that while SC is typically higher in rural areas^(16,17)^, the positive outcomes of SC (e.g. greater fruit consumption) are more prevalent in urban areas, possibly due to more densely populated communities where neighbours have stronger awareness of each other^(11,12)^. While results of this study suggest the opposite relationship is true for network diversity, our results do corroborate that rationale for the civic engagement dimension of SC where individuals who lived in town (i.e. a more densely populated area) were more likely to meet the DGA recommendation for fruit with more civic engagement. Our interest in perceived rurality has more recently been reflected in the literature as a nuanced understanding of residential environments worthy of investigation^(38,40,41)^, as evidenced by the recent creation of the Urbanization Perceptions Small Area Index^(40)^ and the addition of a perceived neighbourhood question to the American Housing Survey^(41)^. However, as this is the only study to our knowledge to explore perceived rurality, SC and eating behaviours, more research is needed to explicate this relationship and compare differences between perceived and observed rurality in relation to SC and healthy eating.

### Demographic factors related to social capital and healthy eating

Contrary to previous evidence that suggests AA/Blacks eat more fruit compared with Whites on both a national and state level^(2,22)^, we found that significantly more White participants in our sample met DGA recommendations for daily fruit intake compared with AA/Black participants. Our findings do support other research that indicates greater SSB consumption among AA/Blacks compared with Whites^(29)^. Additionally, we found significant results for the bivariate analyses exploring the relationships between race and each SC dimension, with Whites experiencing significantly higher levels of each dimension of SC. Further, and aligned with our hypothesis, we found that race/ethnicity moderated the relationship between vegetable consumption and network diversity for Whites but not AA/Blacks. While previous research has not explored how race/ethnicity moderates the SC–healthy eating relationship, our findings support past research on racial disparities related to SC. Previous theories have posited that SC is unevenly distributed to all members of a society, with Whites benefitting more from SC compared with minorities^(24,30,42)^. Specifically, higher levels of civic engagement (e.g. community association membership and voter registration)^(24,42)^ and generalised trust^(43)^ have been found among Whites compared with minorities. Perhaps as people participate in more civic activities, their social networks broaden, which fosters greater levels of trust^(44)^. Additionally, McDonald and Day^(30)^ discussed the way in which SC can create inequalities in the workplace, where Whites, particularly White men, are selected for high-level job openings over women and racial/ethnic minorities despite not searching for those positions, largely due to the information spread through White men’s social networks. Our findings, the first exploring the role of race within the healthy eating–SC relationship, indicate that disparities are present in this relationship where the eating behaviours of Whites benefit more from SC compared with AA/Blacks.

Gender was only associated with SSB consumption in bivariate analyses, whereby a greater percentage of women than men consumed zero SSB per d, which is unsurprising given past research^(5)^. Men, however, had significantly higher levels of social trust, social reciprocity and civic engagement, confirming findings by Eriksson and colleagues^(10)^ yet contradicting those by Pinillos-Franco and Kawachi^(23)^. We also found that gender moderated the relationship between SSB consumption and social trust, where men (but not women) were more likely to consume zero SSB per d with increased social trust. To our knowledge, only one study has explored the relationship between SC and SSB consumption, where a one-unit increase in SC was associated with a 3·6-ounce decrease in daily SSB consumption among a sample of rural Virginians^(20)^. Our results support those findings among men. Nevertheless, as SSB consumption is so rarely included as a healthy eating behaviour examined in relation to SC, it is unclear if the mechanisms thought to link fruit and vegetable consumption to SC (i.e. diffusion of health-promoting knowledge and resources and social control over health-related behaviours) hold true for SSB consumption as well.

### Limitations and future directions

The current study had several limitations. These cross-sectional data prevent us from making causal claims, so it may be that high levels of food insecurity limit one’s ability to cultivate SC rather SC being protective against food insecurity, for instance, or there may be a third variable explaining these associations. Longitudinal and qualitative data could elucidate the causal pathways between SC and healthy eating behaviours. It may be considered a weakness that we cannot compare the amounts of the dimensions of SC in our study to previous research due to the differing conceptions and measures of SC within the field; however, we believe our findings contribute to the understanding of the complex construct of SC. Additionally, these results describe select communities in Georgia and may not generalise to other communities. Further, although these data represent five rural Georgia counties, it should be noted that respondents’ classification of their rurality (i.e. in town *v*. in the country) is subjective; therefore, these findings describe how respondents perceive the degree of rurality of their residence within the rural county in which they reside. Lastly, though we did find several significant relationships, the effect sizes of those relationships were relatively small.

Despite these limitations, this study expands on previous research by gaining a more comprehensive picture of SC^(21)^ and its associations with healthy eating behaviours in rural areas and fills a crucial gap in the literature by exploring contextual and demographic moderators of the SC–healthy eating relationship. At a time when rural residence is so often discussed as a ‘risk factor’ for various conditions^(3,6)^ and with many people choosing to leave rural areas for cities and suburbs, it is important for interventions within rural communities to harness the abundance of SC that exists there. Community gardens are one intervention strategy that may foster SC while also increasing fruit and vegetable consumption^(26)^. Participation in community gardens has the potential to (1) increase dimensions of SC, for example, creating a community-centred activity (i.e. community engagement), producing food resources for community members to share (i.e. social reciprocity), and fostering interactions with new people (i.e. network diversity) and (2) result in improved diet through the food grown, the dissemination of information related to gardening and healthy eating, and the social influence to eat healthfully^(26)^. In fact, healthy eating interventions, particularly those at the interpersonal level of the Social Ecological Model^(45)^, often rely on social networks and social support as change mechanisms^(46,47)^; those mechanisms can be scaled up to the community level, for instance, through group-based interventions, whereby social support fosters social reciprocity and social networks enhance network diversity.

Many organisations (e.g. Robert Wood Johnson Foundation) are investing in rural community efforts to increase SC and impact health behaviours, including healthy eating. Therefore, it is important to understand how SC is related to healthy eating, in which contexts, and for whom, not only to most effectively harness SC, but to ensure that SC and its benefits are equitably distributed. Our findings reveal the complex interplay among dimensions of SC and eating behaviours and highlight the need for qualitative research to illuminate possible causal mechanisms and how they may differ depending on demographic and contextual characteristics.

## References

[1] U.S. Department of Agriculture & U.S Department of Health and Human Services (2020) Dietary Guidelines for Americans, 2020–2025. 9th ed. https://www.dietaryguidelines.gov/sites/default/files/2020-12/Dietary_Guidelines_for_Americans_2020-2025.pdf (accessed August 2021).

[2] Lee-Kwan SH , Moore LV , Blanck HM et al. (2017) Disparities in state-specific adult fruit and vegetable consumption — United States, 2015. MMWR Morb Mortal Wkly Rep 66, 1241–1247. 29145355 10.15585/mmwr.mm6645a1 PMC5726245

[3] Lutfiyya MN , Chang LF & Lipsky MS (2012) A cross-sectional study of US rural adults’ consumption of fruits and vegetables: do they consume at least five servings daily? BMC Public Health 12, 280. 22490063 10.1186/1471-2458-12-280 PMC3365871

[4] Dean WR & Sharkey JR (2011) Food insecurity, social capital and perceived personal disparity in a predominantly rural region of Texas: an individual-level analysis. Soc Sci Med 72, 1454–1462. 21497429 10.1016/j.socscimed.2011.03.015 PMC3090453

[5] Sharkey JR , Johnson CM & Dean WR (2011) Less-healthy eating behaviors have a greater association with a high level of sugar-sweetened beverage consumption among rural adults than among urban adults. Food Nutr Res 55, 5819. 10.3402/fnr.v55i0.5819 PMC3153312 21845142

[6] Lutfiyya MN , McCullough JE , Haller IV et al. (2012) Rurality as a root or fundamental social determinant of health. Dis Mon 58, 620–628. 23062678 10.1016/j.disamonth.2012.08.005

[7] Putnam RD (2000) Bowling Alone: the Collapse and Revival of American Community. (A Touchstone Book). New York: Simon & Schuster.

[8] Johnson CM , Sharkey JR & Dean WR (2010) Eating behaviors and social capital are associated with fruit and vegetable intake among rural adults. J Hunger Environ Nutr 5, 302–315. 21116465 10.1080/19320248.2010.504094 PMC2992326

[9] Poortinga W (2006) Do health behaviors mediate the association between social capital and health? Prev Med 43, 488–493. 16860857 10.1016/j.ypmed.2006.06.004

[10] Eriksson M , Dahlgren L , Janlert U et al. (2010) Social capital, gender and educational level impact on self-rated health. Open Public Health J 3, 1–12.

[11] Ziersch AM , Baum F , Darmawan IGN et al. (2009) Social capital and health in rural and urban communities in South Australia. Aust N Z J Public Health 33, 7–16. 19236353 10.1111/j.1753-6405.2009.00332.x

[12] Nummela O , Sulander T , Rahkonen O et al. (2008) Social participation, trust and self-rated health: a study among ageing people in urban, semi-urban and rural settings. Health Place 14, 243–253. 17686647 10.1016/j.healthplace.2007.06.006

[13] Nosratabadi S , Khazami N , Abdallah MB et al. (2020) Social capital contributions to food security: a comprehensive literature review. Foods 9, 1650. 33198127 10.3390/foods9111650 PMC7698312

[14] Kawachi I & Berkman L (2000) Social cohesion, social capital, and health. Soc Epidemiol 174, 290–319.

[15] Chen WL , Zhang CG , Cui ZY et al. (2019) The impact of social capital on physical activity and nutrition in China: the mediating effect of health literacy. BMC Public Health 19, 1713. 31856789 10.1186/s12889-019-8037-x PMC6924071

[16] Sørensen JFL (2016) Rural–urban differences in bonding and bridging social capital. Reg Stud 50, 391–410.

[17] Hofferth SL & Iceland J (1998) Social capital in rural and urban communities1. Rural Sociol 63, 574–598.

[18] Yip W , Subramanian SV , Mitchell AD et al. (2007) Does social capital enhance health and well-being? Evidence from rural China. Soc Sci Med 64, 35–49. 17029692 10.1016/j.socscimed.2006.08.027

[19] Buck-McFadyen E , Isaacs S , Strachan P et al. (2018) How the rural context influences social capital: experiences in two Ontario communities. J Rural Community Dev 14, 1–18.

[20] Bailey A (2016) Exploring health disparities in rural regions of Virginia: the Impact of Health Literacy and Social Capital. Doctoral Dissertation, Virginia Polytechnic Institute and State University. VTechWorks. https://vtechworks.lib.vt.edu/bitstream/handle/10919/78318/Bailey_AN_D_2016.pdf?sequence=1 (accessed August 2021).

[21] Gilbert KL , Quinn SC , Goodman RM et al. (2013) A meta-analysis of social capital and health: a case for needed research. J Health Psychol 18, 1385–1399. 23548810 10.1177/1359105311435983 PMC4236001

[22] Grimm KA , Foltz JL , Blanck HM et al. (2012) Household income disparities in fruit and vegetable consumption by state and territory: results of the 2009 behavioral risk factor surveillance system. J Acad Nutr Diet 112, 2014–2021. 23174688 10.1016/j.jand.2012.08.030

[23] Pinillos-Franco S & Kawachi I (2018) The relationship between social capital and self-rated health: a gendered analysis of 17 European countries. Soc Sci Med 219, 30–35. 30359904 10.1016/j.socscimed.2018.10.010

[24] Hero RE (2003) Social capital and racial inequality in America. Perspect Polit 1, 113–122.

[25] Coleman-Jensen A , Rabbitt MP , Gregory CA et al. (2021) Household Food Security in the United States in 2020. Washington, DC: U.S. Department of Agriculture, Economic Research Service.

[26] Martin G , Clift R & Christie I (2016) Urban cultivation and its contributions to sustainability: nibbles of food but oodles of social capital. Sustainability 8, 409.

[27] Drisdelle C , Kestens Y , Hamelin AM et al. (2020) Disparities in access to healthy diets: how food security and food shopping behaviors relate to fruit and vegetable intake. J Acad Nutr Diet 120, 1847–1858. 32593668 10.1016/j.jand.2020.03.020

[28] Hanson KL & Connor LM (2014) Food insecurity and dietary quality in US adults and children: a systematic review. Am J Clin Nutr 100, 684–692. 24944059 10.3945/ajcn.114.084525

[29] Dai J , Soto MJ , Dunn CG et al. (2021) Trends and patterns in sugar-sweetened beverage consumption among children and adults by race and/or ethnicity, 2003–2018. Public Health Nutr 24, 2405–2410. 33843567 10.1017/S1368980021001580 PMC10195631

[30] McDonald S & Day JC (2010) Race, gender, and the invisible hand of social capital. Sociol Compass 4, 532–543.

[31] Resnicow K , Odom E , Wang T et al. (2000) Validation of three food frequency questionnaires and 24-hour recalls with serum carotenoid levels in a sample of African-American Adults. Am J Epidemiol 152, 1072–1080. 11117617 10.1093/aje/152.11.1072

[32] Britten P , Marcoe K , Yamini S et al. (2006) Development of food intake patterns for the MyPyramid food guidance system. J Nutr Educ Behav 38, S78–92. 17116598 10.1016/j.jneb.2006.08.007

[33] Park S & Pan L (2013) A Data User’s Guide to the BRFSS Sugar-Sweetened Beverage Questions: How to Analyze Consumption of Sugar-Sweetened Beverages. Atlanta, GA: Behavioral Risk Factor Surveillance System, Centers for Disease Control and Prevention. https://www.cdc.gov/brfss/data_documentation/pdf/brfss_ssb-userguide.pdf (accessed February 2021).

[34] Roper Center for Public Opinion Research (2001) Social Capital Community Benchmark Survey: Methodology and Documentation. University of Connecticut. https://ropercenter.cornell.edu/misc/usmisc2000-soccap/usmisc2000-soccap.PDF (accessed August 2021).

[35] Sampson RJ , Raudenbush SW & Earls F (1997) Neighborhoods and violent crime: a multilevel study of collective efficacy. Science 277, 918–924. 9252316 10.1126/science.277.5328.918

[36] Martin KS , Rogers BL , Cook JT et al. (2004) Social capital is associated with decreased risk of hunger. Soc Sci Med 58, 2645–2654. 15081212 10.1016/j.socscimed.2003.09.026

[37] Engbers TA , Thompson MF & Slaper TF (2017) Theory and measurement in social capital research. Soc Indic Res 132, 537–558.

[38] Kegler MC , Alcantara I , Haardörfer R et al. (2014) The influence of home food environments on eating behaviors of overweight and obese women. J Nutr Educ Behav 46, 188–196. 24809866 10.1016/j.jneb.2014.01.001

[39] Njai R , Siegel P , Yin S et al. (2017) Prevalence of perceived food and housing security — 15 states, 2013. MMWR Morb Mortal Wkly Rep 66, 12. 28081062 10.15585/mmwr.mm6601a2 PMC5687269

[40] Bucholtz S , Molfino E & Kolko J (2020) *The Urbanization Perceptions Small Area Index: An Application of Machine Learning and Small Area Estimation to Household Survey Data*. Washington, DC: US Department of Housing and Urban Development. https://www.huduser.gov/portal/AHS-neighborhooddescription-study-2017.html#small-area-tab (accessed February 2022).

[41] Bucholtz S & Kolko J (2018) America Really is a Nation of Suburbs. Bloom CityLab. https://www.citylab.com/life/2018/11/data-most-american-neighborhoods-suburban/575602/ (accessed March 2022).

[42] Foster-Bey J (2008) Do Race, Ethnicity, Citizenship and Socio-economic Status Determine Civic-Engagement? CIRCLE, Working Paper #62. https://files.eric.ed.gov/fulltext/ED505266.pdf (accessed February 2022).

[43] Fairbrother M & Martin IW (2013) Does inequality erode social trust? Results from multilevel models of US states and counties. Soc Sci Res 42, 347–360. 23347481 10.1016/j.ssresearch.2012.09.008

[44] Glanville JL (2016) Why does involvement in voluntary associations promote trust? Examining the role of network diversity. Sociol Inq 86, 29–50.

[45] McLeroy KR , Bibeau D , Steckler A et al. (1988) An ecological perspective on health promotion programs. Health Educ Q 15, 351–377. 3068205 10.1177/109019818801500401

[46] Smith ML , Lee S , Towne SD Jr et al. (2020) Impact of a behavioral intervention on diet, eating patterns, self-efficacy, and social support. J Nutr Educ Behav 52, 180–186. 31540863 10.1016/j.jneb.2019.06.008

[47] de la Haye K , Bell BM & Salvy SJ (2019) The role of maternal social networks on the outcomes of a home-based childhood obesity prevention pilot intervention. J Soc Struct 20, 7–28. 31827412 10.21307/joss-2019-004 PMC6905644

